# ChIP on SNP-chip for genome-wide analysis of human histone H4 hyperacetylation

**DOI:** 10.1186/1471-2164-8-322

**Published:** 2007-09-14

**Authors:** Jennifer A McCann, Enrique M Muro, Claire Palmer, Gareth Palidwor, Christopher J Porter, Miguel A Andrade-Navarro, Michael A Rudnicki

**Affiliations:** 1 Ottawa Health Research Institute, Regenerative Medicine Program, 501 Smyth Road, Ottawa, Ontario, K1H 8L6, Canada; 2 Faculty of Health Sciences, McMaster University Hamilton, Ontario, L8S 4K1, Canada

## Abstract

**Background:**

SNP microarrays are designed to genotype Single Nucleotide Polymorphisms (SNPs). These microarrays report hybridization of DNA fragments and therefore can be used for the purpose of detecting genomic fragments.

**Results:**

Here, we demonstrate that a SNP microarray can be effectively used in this way to perform chromatin immunoprecipitation (ChIP) on chip as an alternative to tiling microarrays. We illustrate this novel application by mapping whole genome histone H4 hyperacetylation in human myoblasts and myotubes. We detect clusters of hyperacetylated histone H4, often spanning across up to 300 kilobases of genomic sequence. Using complementary genome-wide analyses of gene expression by DNA microarray we demonstrate that these clusters of hyperacetylated histone H4 tend to be associated with expressed genes.

**Conclusion:**

The use of a SNP array for a ChIP-on-chip application (ChIP on SNP-chip) will be of great value to laboratories whose interest is the determination of general rules regarding the relationship of specific chromatin modifications to transcriptional status throughout the genome and to examine the asymmetric modification of chromatin at heterozygous loci.

## Background

Chromatin immunoprecipitation (ChIP) is a technique widely used to study interactions of proteins with specific genomic regions [1]. Several methodologies have been devised for the detection of the genomic fragments generated by a ChIP experiment (reviewed in [2]). In particular, the use of DNA microarray methodology (ChIP-on-chip) allows for high-throughput analysis of thousands of genomic sequences simultaneously [3]. Genome tiling arrays covering entire genomes [4] can be used to map the sites of DNA-protein interaction on a genomic scale, although at a high cost.

Here, we propose the use of SNP microarrays (SNP-chip) to evaluate ChIP products (ChIP on SNP-chip) as an alternative to genome tiling arrays. SNP microarrays are designed to genotype thousands of Single Nucleotide Polymorphisms (SNPs) by hybridization of genomic fragments to an array of short nucleotide sequences [5]. The evaluation of SNP-chip hybridization in binary terms (a probe in the SNP-chip either does or does not hybridize) might be appropriate for ChIP-on-chip experiments where a large number of DNA-protein interaction sites have to be detected and the evaluation of the strength of each interaction is not an issue. The mapping of histone modifications throughout the genome fulfills these requirements. To illustrate the ChIP on SNP-chip methodology, we present here a genome-wide analysis of histone H4 hyperacetylation in human myoblasts and myotubes.

Histone H4 hyperacetylation has been associated with increased gene expression [6], and, although the molecular basis of this effect is under investigation [7], the precise pattern of histone acetylation and its effect on gene expression is not completely understood [8]. The assembly of an active transcriptional complex at the promoter is an essential feature of eukaryotic gene expression [9,10]. Histone acetylation of the promoter precedes the activation of many genes and is thought to establish a chromatin environment suitable for the assembly of the transcriptional complex [11,12]. The genomic distribution of H4 acetylation has been studied for individual genes (reviewed in [2]), across human chromosomes 21 and 22 [13], and at whole genome level in yeast [14], but never for a complete mammalian genome. Quantification of the pattern of histone H4 hyperacetylation in the human genome will add valuable information about the mechanisms by which this histone modification affects chromatin structure and controls gene expression.

The analysis of our results indicates significant clusters of H4 hyperacetylation at a range of 300 kilobases in human samples from both myoblasts and myotubes. Complementary analysis of gene expression of the same samples indicates that histone H4 hyperacetylation is positively associated with gene expression at a range of [-300 Kb, +300 Kb] from the start of gene transcription; a much greater range than previously reported. These results show that ChIP on SNP-chip can be used to provide biological insight into how histone H4 hyperacetylation affects both eukaryotic transcription and chromatin's structural integrity.

## Results

**Figure 1:**
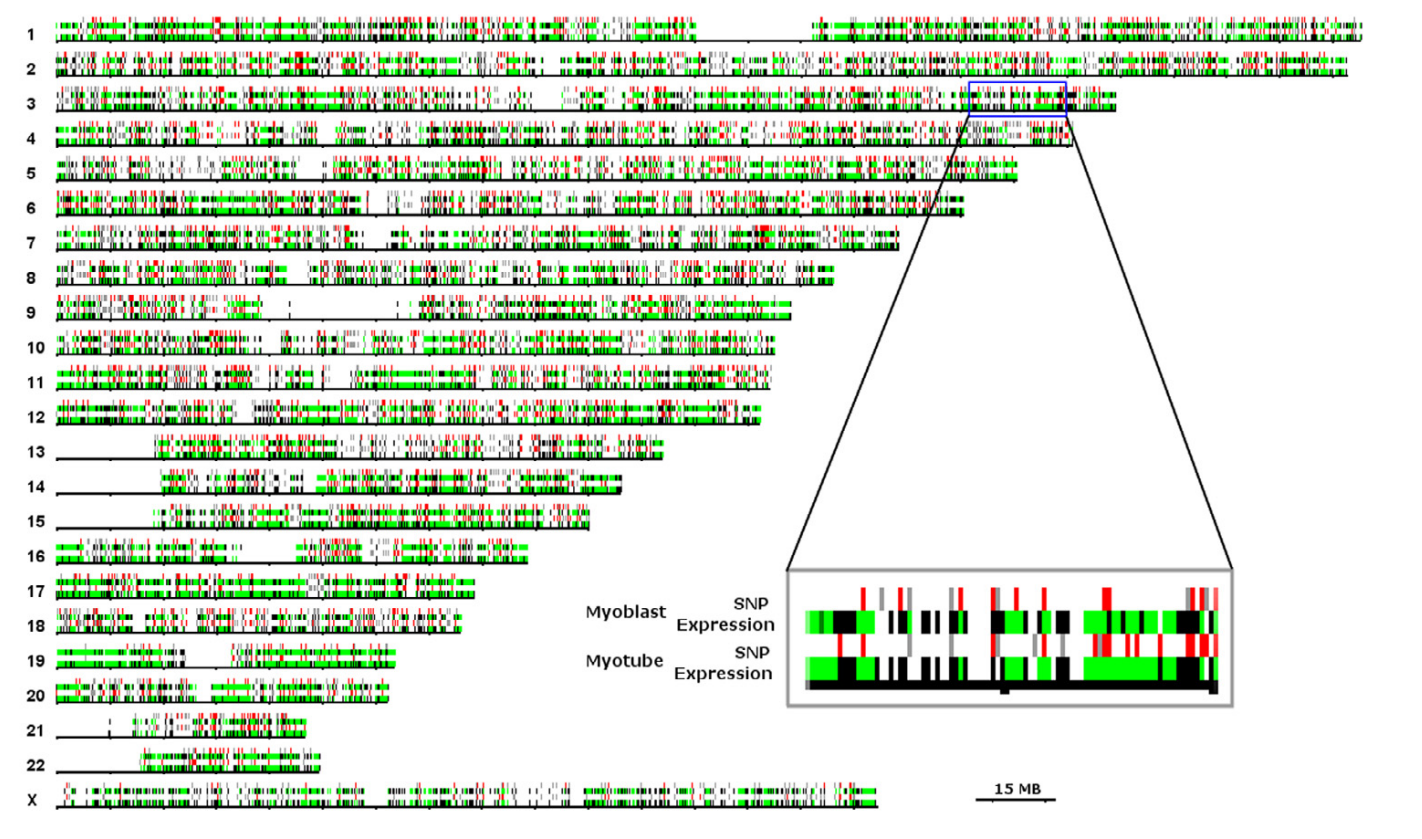
**SNP-array and gene expression DNA microarray data mapped to chromosomal positions**. Positions corresponding to the transcripts detected by the Affymetrix HGU133A/B expression microarray are plotted in green if they were detected and black if they were not detected; corresponding genomic positions of the Affymetrix 10 K SNP-Array probes are plotted in red if they hybridized to DNA in the ChIP on SNP-chip experiment (indicating the position of hyperacetylated histone H4), and grey if not. Ambiguous data points are not shown. All 22 autosomes and the X chromosome are represented. The Y chromosome is not considered in the SNP-Array used. Mb: megabases; SNP: Single Nucleotide Polymorphism.

We assessed the genomic distribution of hyperacetylated histone H4 by ChIP with an antibody specific to the N-terminal tail of histone H4 acetylated at residues Lys5, Lys8, Lys12, and Lys16. Hybridization of the ChIP products to the Affymetrix 10 K SNP microarray produced reproducible results for 3,914 probes (1,817 hybridized and 2,097 not hybridized) in a sample of human myoblasts, and for 4,897 probes (2,510 hybridized and 2,387 not hybridized) in a sample of human myotubes (see Methods). An overview of the genomic distribution of these results is given in Figure 1.

**Figure 2:**
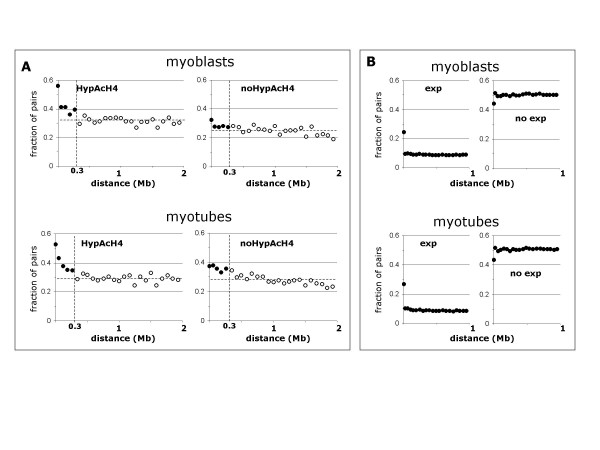
**Genomic correlation of histone H4 hyperacetylation and gene expression**. (A) For all pairs of SNP-array probes detecting genomic sites separated a given distance range, the plots show (for myoblasts (top) and myotubes (bottom)) the fraction of pairs where both probes are hybridized (left), and both probes are not hybridized (right). For pairs of SNP-probes at distances below 0.3 Mb (full circles) the fraction of those where both detect histone H4 hyperacetylation is significantly above the average for those at larger distances (horizontal discontinuous line). Each data point was computed from all the SNP-probe pairs at the corresponding distance range (more than 500 and 700 for myoblasts and myotubes, respectively). The data is provided as Supplementary Table S1. (B) For all pairs of Affymetrix DNA microarray HGU133A/B probesets detecting transcripts with transcription starts at a given distance range, the plot shows (for myoblasts (top) and myotubes (bottom)) the fraction of pairs where both probesets are hybridized (left), and both probesets are not hybridized (right). Note that the Affymetrix DNA microarray HGU133A/B includes often multiple probesets for the same transcript or for alternative splice variants of the same transcript that start at the same position; these probesets explain the higher than random values for the gene expression plot at near-zero distance. All data points were computed using all the pairs of probesets at the distance range, which were more than 30,000 except for the lowest range point (pairs at less than 2 Kb; 7,093 probeset pairs). The data is provided as Supplementary Table S2. Apart from this sort range effect, which is related to probeset design, no other effect can be observed indicating any correlation of gene expression between proximal genes. HypAcH4: hyperacetylated histone H4; Mb: megabases; exp: expressed genes; no exp: not expressed genes.

Analysis of the hybridization reported by SNP-array probes indicates that there is a tendency for probes detecting H4 hyperacetylation to cluster in nearby genomic positions (Figure 2A, Additional file 1). This tendency is significant up to a range of 300 kb meaning that histone H4 hyperacetylation occurs in clusters that can span 300 kb. In contrast, non-hybridized probes do not cluster (Figure 2A). It is unlikely that the clustering effect is due to ChIP'd DNA fragments hybridizing to multiple probes in the SNP-array, since the DNA is digested with XbaI, which cuts once every 4 kb in the human genome, on average.

To study the relationship between histone H4 hyperacetylation and gene expression, we obtained gene expression data from equivalent human myoblast and myotube samples. Analysis of mRNA cellular transcripts using the Affymetrix HGU133A/B chip set produced reproducible results for 38,865 probesets (10,872 hybridized and 25,993 not hybridized) in human myoblasts, and for 36,905 probesets (10,814 hybridized and 26,091 not hybridized) in human myotubes. An overview of the genomic distribution of these results is given in Figure 1.

In contrast to our observation for the SNP-array probes, we did not observe correlation between probesets corresponding to transcripts in neighbouring regions of the genome (Figure 2B, Additional file 2). This is consistent with multiple evidence showing the general lack of gene expression correlation between neighbouring genes in eukaryotic genomes with few exceptions such as the histone genes or Hox genes (see e.g. [15]). Therefore, the clusters of hyperacetylated H4 that were identified cannot be explained by the correlated expression of clusters of genes.

**Figure 3:**
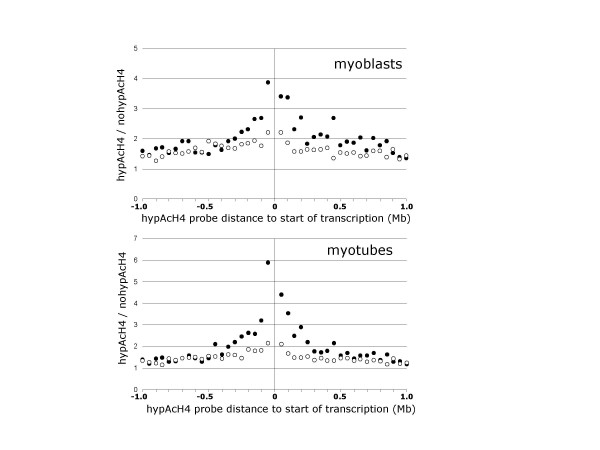
**Association between histone H4 hyperacetylation and gene expression in human myoblasts and myotubes**. For all pairs of SNP-array probes and gene transcription starts at a given distance range, the plot shows the ratio of hybridizing over non-hybridizing SNP-array probes, for genes detected as transcribed (filled circles), or not transcribed (empty circles). The centre of the graph indicates the start of transcription for a gene with negative distances representing the upstream region and positive distances representing the downstream region. The detection of histone H4 modification is markedly higher in the [-0.3 Mb, +0.3 Mb] distance range of the start of transcription of expressed genes indicating a positive association between histone H4 hyperacetylation and gene expression. Each data point is computed from all pairs of SNP-Array probe to DNA microarray probeset at the given distance range, always more than 100. The data is provided as Supplementary Table S3. HypAcH4: hyperacetylated histone H4; noHypAcH4: non-hyperacetylated histone H4; Mb: megabases.

Next, we studied how the distribution of hybridization values reporting histone H4 hyperacetylation near the start of transcription of genes is modified by the status of expression of the gene. Indeed, we observed that histone modification was significantly higher around the start of transcription of expressed genes than non-expressed genes, both in human myoblasts and myotubes, with a somewhat larger effect upstream the transcription start site than downstream (Figure 3, Additional File 3). We identified a significant effect at a distance of 150 kilobases from the start of transcription. At this range, in myoblasts the ratio of hybridizing to non-hybridizing SNP-probes was 2,350/792 = 2.96 for expressed genes, and of 5,514/2,890 = 1.91 for non-expressed genes (Chi square of 88). Similarly, in myotubes, the ratio of hybridizing to non-hybridizing SNP-probes was 2,995/883 = 3.39 for expressed genes, and of 6,741/3,718 = 1.81 for non-expressed genes (Chi square of 214). Both value distributions are significant at P value < 0.0001 according to a Chi-square test. Statistically significant, though less marked, differences were observed at distances up to 300 kilobases (see Figure 3).

Imprinted loci should display allele specific distribution of specific chromatin modifications. Therefore, we examined all heterozygous loci following determination of genotypes by SNP analysis of total DNA isolated from myoblasts to identify novel regions displaying reciprocally modified chromatin. Of the SNPs assessed, 2,024 SNPs were found to be heterozygous. 408 and 563 of the heterozygous SNPs had a relative signal above background in myoblasts and in myotubes, respectively. The contribution of each allele to the overall relative signal was equivalent in the majority of these heterozygote SNPs. However, 137 of the 408 and 67 of the 496 heterozygous SNPs had signal from only one allele in myoblasts and myotubes, respectively. In most of the 248 heterozygous SNPs with signal both in myoblasts and in myotubes, the relative contribution of each allele was maintained between the myotube and myoblast samples (20 and 188 with signal from one or two alleles, respectively). However, the other 40 SNPs suggested a change (39 of them from two alleles to one, and just 1 from one allele to the other). Together, these data indicate an unanticipated level of complexity in chromatin structure acetylation present at numerous heterozygous loci.

## Discussion and conclusion

We have presented the novel use of SNP-arrays for identification of DNA fragments in a chromatin immunoprecipitation experiment (ChIP on SNP-chip). Our application of this methodology to the analysis of histone H4 hyperacetylation in human myoblasts and myotubes suggests that this histone modification happens in clusters across large genomic distances (possibly up to 300 kilobases).

The study of gene expression in equivalent samples indicates no correlation of gene expression in nearby genes, but that the clusters of hyperacetylated histone H4 are associated with expressed genes at ranges of up to 300 kilobases. These results together suggest that histone H4 hyperacetylation is a relatively imprecise mechanism that acts over genomic regions sometimes spanning multiple genes, that are then expressed or not according to the more precise activity of the transcription factor machinery. It has been suggested that such a mechanism could facilitate rapid gene transcription in response to external stimuli [16].

We have demonstrated that ChIP on SNP-chip can be used to characterize genomic sites of DNA-protein interaction. ChIP on SNP-chip makes it feasible to establish general rules of association between proteins and DNA but does not provide precise information regarding the strength of individual interaction sites because of the intrinsic noise level of the SNP-array used in this way. We recommend this methodology for experiments like the one presented in this manuscript, where the aim is to identify a large number of genomic sites of protein-DNA interaction. In particular, this methodology is ideally suited to the study of histone modifications. In addition, the use of SNP chips provides information about the allelic distribution of the chromatin modifications under study. This could be of value if the researcher is studying imprinting or alternatively the effect genetic variants on the binding of a protein to DNA. Our analysis of heterozygous loci suggest an unanticipated level of complexity in chromatin acetylation between alleles. Since we performed the analyses presented in this manuscript, newer SNP arrays with higher resolution have been produced. For example, the new Genome-Wide Human SNP Array 6.0 (Affymetrix) features more than 906,600 single nucleotide polymorphisms (SNPs) and more than 946,000 probes for the detection of copy number variation. Without question, the new generation SNP arrays allow thorough coverage of the genome for efficient analysis of ChIP products.

## Methods

### Cell Culture

Human fetal skeletal myoblasts (a generous gift from Dr. Eric Shoubridge) were cultured in SKGM-2 media (Cat. # cc-3245, Cambrex, East Rutherford, NJ) containing 10% fetal bovine serum (Hyclone, Logan, Utah) 10 ng/ml human epidermal growth factor, 0.1 mg/mL insulin, 0.5 mg/mL BSA, 0.5 mg/mL fetuin, 0.39 μg/mL dexamethasone and 50 μg/mL gentamicin and maintained at 37°C and 5% CO2. Cells were grown to approximately 70% confluence and were induced to differentiate in to multinucleated myotubes by mitogen withdrawal. Cells were maintained in differentiation media (DMEM supplemented with 2.5% horse serum) for five days prior to harvesting.

### RNA extraction and hybridization

Total RNA was extracted from 1 × 10^6 ^myoblasts and differentiated myotubes using the RNeasy Mini Kit (Qiagen, Valencia, CA (5 μg) according to the manufacturer's instructions. RNA was quantified, quality-checked by the Bioanalyzer (Agilent) and reverse-transcribed with a cDNA synthesis kit in the presence of SuperScript II RT (Invitrogen-Life Technologies, Inc.) and an oligo dT-T7 primer (Affymetrix Inc., Santa Clara, CA). Ten microliters of purified cDNA were used for the in vitro transcription (IVT) amplification reaction, in the presence of biotinylated nucleotides (Enzo Biochem Inc.). Labeled cRNA (15 μg) was fragmented by incubation at 94°C for 35 min in fragmentation buffer (GeneChip Sample Cleanup, Qiagen) and hybridized competitively against the Affymetrix HG-U133 microarray set. Arrays were scanned using a GeneArray 2500 scanner (Affymetrix) and analyzed using MicroArray Suite 5.0 (Affymetrix).

### DNA extraction and chromatin immunoprecipitation

Total genomic DNA was extracted from myoblasts using the Qiagen DNeasy Tissue Kit (Valencia CA) and was used as a control for the SNP-Array studies. Antibody used in Chromatin immunoprecipitation was anti-acetyl-Histone H4 peptide corresponding to amino acids 2–19 of *Tetrahymena *histone H4 acetylated on Lys5, Lys8, Lys12 and Lys16 (Upstate Cat #06–866). Chromatin immunoprecipitation was performed using the ChIP Assay kit from Upstate Biotech (Charlottesville, VA) (Cat# 17–295) as per manufacturer's instructions. Briefly, 1 × 10^8 ^cells were cross-linked for 15 minutes at room temperature with 1% formaldehyde. Cells were washed twice with ice-cold PBS, scraped from tissue culture plates and subsequently resuspended in SDS lysis buffer (1% SDS, 10 mM EDTA, 50 mM Tris-HCl, pH8.1) supplemented with protease inhibitors (Mini Complete, Roche, Cat. # 1836153) and lysed for 10 minutes on ice. DNA was sheared to between 1 and 2 kb by sonication for three 15-second pulses with a 1.5 mm step probe equipped sonicator set to a magnitude of 30%. Sonicated lysates were cleared by centrifugation for 10 minutes at 13,000 rpm at 4°C. Cleared lysates were diluted 10 fold in 0.01% SDS, 1.1% Triton ×-100, 1.2 mM EDTA, 16.7 mM Tris HCl, pH8.1, 167 mM NaCl and incubated with 70 μl of Salmon Sperm DNA/Protein A Agarose-50% slurry (Upstate Biotech, Cat# 16–157C) to reduce non-specific background for 1 hour at 4°C with rotation. Five μls of Anti-acetyl-H4 (Upstate Biotech, Cat. # 06–866) was added to the lysates (per 10^6^ cell equivalents). Immune complexes were recovered with Salmon Sperm DNA/Protein A Agarose and washed twice with low salt buffer (0.1% SDS, 1% Triton ×-100, 2 mM EDTA, 20 mM Tris HCl, pH8.1, 150 mM NaCl); twice with high salt buffer (0.1% SDS, 1% Triton ×-100, 2 mM EDTA, 20 mM Tris HCl, pH 8.1, 500 mM NaCl); twice with LiCl buffer (0.25 M LiCl, 1% NP40, 1% deoxycholate, 1 mM EDTA, 10 mM Tris HCl, pH 8.1), and twice with TE. Immune complexes were eluted with an elution buffer containing 1% SDS and 0.1 M NaHCO3 for 30 min at room temperature with rotation. DNA-protein crosslinks were reversed for 4 hrs at 65°C with 0.2 mM NaCl. Samples were deproteinated and DNA isolated using columns as described by the manufacturer (Qiagen). DNA was eluted from columns with 30 μls of ddH_2_O.

### Target Preparation

Immunoprecipitated DNA and control (whole-cell extract) DNA were assayed according to the protocol (GeneChip Mapping Assay manual) supplied by Affymetrix. Briefly, a total of 250 ng DNA was digested with XbaI then ligated to XbaI adaptor before PCR amplification. Cycling conditions were: 95°C for 3 minutes followed by 35 cycles of 95°C for 20 seconds, 59°C for 15 seconds, and 72°C for 15 seconds. Final extension was done at 72°C for 7 minutes (DNA Engine Tetrad PTC-225, MJ Research, Waltham, MA). To evaluate PCR products, 3 μL of each PCR product was mixed with 3 μL of 2× gel loading dye on 2% Tris-borate EDTA gel and run at 120 V for 1 hour to check for the expected product between 250 and 1,000 bp. Twenty μg of PCR product was fragmented with DNase I and biotinylated overnight at 37°C using biotin-N6-ddATP (Perkin Elmer) and terminal transferase (Promega, Madison WI). Target hybridization, washing, scanning and staining were performed as recommended by the manufacturer.

### Genotype generation

The 10 K SNP arrays were scanned with the Affymetrix GeneChip Scanner 3000 using GeneChip Operating System 1.0 (Affymetrix). Data files were generated automatically and genotype calls were made automatically by GeneChip DNA Analysis Software 2.0 (Affymetrix). Genomic location of SNP probesets was derived using the Affymetrix GeneChip Mapping 10 K library file (Mapping10K_Xba131) and the Ensembl human genome map (v27.35a.1), based on the release 35 of the human genome sequence (May 2004). Each probeset is identified in the Affymetrix annotation by its tscID (The SNP Consortium). These IDs are mapped to rsIDs (NCBI RefSNP ID), which are used to identify SNP positions in the Ensembl database.

### SNP-Array probeset selection and analysis

This study used a pre-commercial release version of the Affymetrix GeneChip Mapping 10 K SNP Array for the human genome (ax13339). This microarray contains probesets for 10,043 SNPs distributed across the 22 autosomes and the X chromosome. An initial selection process identified probesets that map to an unambiguous location in the human genome; tscIDs were mapped to rsIDs, for which genomic locations can be found in Ensembl. Screening removed 448 tscIDs, which could not be mapped to any rsID; 2,362 rsIDs not localized in Ensembl; and 33 with multiple locations. The remaining 7,200 probesets, which have a unique location, were used in our analysis.

As a further selection for high quality SNP data, we performed a control genotyping experiment in triplicate using the total DNA from the same myoblast cell line. Of the 7,200 uniquely mapped probesets, only the 6,464 for which the same genotyping was obtained in at least two of the three replicates were used for our analysis.

The analysis of SNP-array data was performed in biological triplicates. We analyzed the results from SNP arrays using the Genotyping Tools V 1.0 (Affymetrix). This tool assesses whether measurable hybridization to a probeset is present, and provides heterozygosity data. Probes were classified as hybridizing or not based on calls of Present (P) (present in at least two of three replicates) or Absent (A) (absent in at least two of three replicates). All other probesets were not included in further analysis.

### DNA microarray probeset selection and analysis

The Affymetrix HGU133A/B gene expression array set contains 44,760 probesets from genes on the 22 autosomes and on chromosomes X and Y. We selected the probesets that were unambiguously mapped to a genomic position in the NetAffx probeset annotations [17] (April 12, 2005, , based on NCBI release 35 of the human genome sequence). Screening removed 858 probesets without a genomic location in NetAffx, 3,114 with multiple locations, and 52 corresponding to genes in chromosome Y, which is not covered by the SNP Array. The remaining 40,736 probesets with a unique genomic location were used in our analysis.

We analyzed the results from the expression arrays using the MicroArray Suite 5.0, (Affymetrix). This tool assesses whether measurable hybridization to a probeset is present. Analysis of gene expression was performed in biological triplicates. Probesets were classified as hybridizing or not based on calls of Present (P) (present in at least two of three replicates) or Absent (A) (absent in at least two of three replicates), respectively. All other probesets were not included in further analysis.

### Availability of microarray data in GEO

We have deposited all microarray data used in this work at the Gene Expression Omnibus database (National Center for Biotechnology Information) where it is available under the super-series identifier GSE4133. This super series is composed of the following subset series: GSE4131 (triplicate Affymetrix HGU133A/B expression chips hybridized to RNA extracted from myoblasts and myotubes) and GSE4132 (triplicate Affymetrix 10 K ax 13339 SNP-chips were hybridized to DNA from myoblasts (control), myoblast ChIP, and myotube ChIP). The gene expression data is also available at the StemBase database of stem cell gene expression data [18] under experiment identifier E204 (containing sample S267 for the myoblasts, and sample S273 for the myotubes).

## Authors' contributions

JAM and CP performed the cell and molecular aspects of the experiments and drafted the manuscript. JY carried out the immunoassays. EMM, GP, and CJP designed and wrote the programs to perform the bioinformatics analysis. MAA participated in the design of the study and helped draft the manuscript. MAR conceived the study, and participated in its design and coordination and helped to draft the manuscript. All authors read and approved the final manuscript.

## Supplementary Material

Additional file 1 **Supplementary Table S1**. Genomic correlation of histone H4 hyperacetylation. For all pairs of SNP-array probes detecting genomic sites separated a given distance range, the table shows the numbers of pairs according to their detection status. Columns are: distance, upper limit of distance range (in bp); total_blast, number of pairs at the distance range for the myoblast sample; aa_blast, fraction of pairs where both probes did not detect histone H4 hyperacetylation in the myoblast sample; pp_blast, fraction of pairs where both probes detected histone H4 hyperacetylation in the myoblast sample; total_tube, number of pairs at the distance range for the myotube sample; aa_tube, fraction of pairs where both probes did not detect histone H4 hyperacetylation in the myotube sample; pp_tube, fraction of pairs where both probes detected histone H4 hyperacetylation in the myotube sample. This data was used for the graphs in Figure 2A. Click here for file

Additional file 2 **Supplementary Table S2**. Genomic correlation gene expression. For all pairs of Affymetrix DNA microarray HGU133A/B probesets detecting transcripts with transcription starts at a given distance range, the table shows the number of pairs according to their detection status. Columns are: distance, upper limit of distance range (in bp); total_blast, number of pairs at the distance range for the myoblast sample; aa_blast, fraction of pairs where both probesets did not detect gene expression in the myoblast sample; pp_blast, fraction of pairs where both probesets detected gene expression in the myoblast sample; total_tube, number of pairs at the distance range for the myotube sample; aa_tube, fraction of pairs where both probesets did not detect gene expression in the myotube sample; pp_tube, fraction of pairs where both probes detected gene expression in the myotube sample. This data was used for the graphs in Figure 2B. Click here for file

Additional file 3 **Supplementary Table S3**. Association between histone H4 hyperacetylation and gene expression in human myoblasts and myotubes. For all pairs of SNP-array probes and gene transcription starts at a given distance range, the table shows the number of pairs of SNP-array probes and DNA microarray probesets according to their detection status. Columns are: distance, upper limit of distance range (in bp); pT_blast, number of pairs where the SNP-array probe detected histone H4 hyperacetylation and the DNA microarray probeset detected gene expression in the myoblast sample; pS_blast, number of pairs where the SNP-array probe detected histone H4 hyperacetylation and the DNA microarray probeset did not detect gene expression in the myoblast sample; aT_blast, number of pairs where the SNP-array probe did not detect histone H4 hyperacetylation and the DNA microarray probeset detected gene expression in the myoblast sample; aS_blast, number of pairs where the SNP-array probe did not detect histone H4 hyperacetylation and the DNA microarray probeset did not detect gene expression in the myoblast sample; pT_tube, number of pairs where the SNP-array probe detected histone H4 hyperacetylation and the DNA microarray probeset detected gene expression in the myotube sample; pS_tube, number of pairs where the SNP-array probe detected histone H4 hyperacetylation and the DNA microarray probeset did not detect gene expression in the myotube sample; aT_tube, number of pairs where the SNP-array probe did not detect histone H4 hyperacetylation and the DNA microarray probeset detected gene expression in the myotube sample; aS_tube, number of pairs where the SNP-array probe did not detect histone H4 hyperacetylation and the DNA microarray probeset did not detect gene expression in the myotube sample. This data was used for the graphs in Figure 3. Click here for file
