# Supplementary material for: ChIP on SNP-chip for genome-wide analysis of human histone H4 hyperacetylation
Source: BMC Genomics. 2007 Sep 14;8:322. doi: 10.1186/1471-2164-8-322 (PMC2194786; doi:10.1186/1471-2164-8-322)
Supplement: Additional file 1 — Supplementary Table S1. Genomic correlation of histone H4 hyperacetylation. For all pairs of SNP-array probes detecting genomic sites separated a given distance range, the table shows the numbers of pairs according to their detection status. Columns are: distance, upper limit of distance range (in bp); total_blast, number of pairs at the distance range for the myoblast sample; aa_blast, fraction of pairs where both probes did not detect histone H4 hyperacetylation in the myoblast sample; pp_blast, fraction of pairs where both probes detected histone H4 hyperacetylation in the myoblast sample; total_tube, number of pairs at the distance range for the myotube sample; aa_tube, fraction of pairs where both probes did not detect histone H4 hyperacetylation in the myotube sample; pp_tube, fraction of pairs where both probes detected histone H4 hyperacetylation in the myotube sample. This data was used for the graphs in Figure 2A. [file 1471-2164-8-322-S1.pdf]

Supplementary Table S1

distance	total_blast	aa_blast	pp_blast	total_tube	aa_tube	pp_tube
500	507	0.325	0.562	747	0.373	0.526
53900	500	0.276	0.414	782	0.379	0.432
114200	500	0.272	0.412	737	0.357	0.376
188000	501	0.281	0.359	818	0.331	0.351
266900	500	0.272	0.394	801	0.356	0.347
347400	500	0.282	0.296	856	0.343	0.289
430300	501	0.271	0.353	780	0.3	0.327
499600	500	0.238	0.326	737	0.309	0.318
586700	500	0.246	0.306	802	0.286	0.291
667700	500	0.29	0.314	794	0.322	0.278
752700	500	0.258	0.336	808	0.303	0.29
835600	500	0.254	0.334	792	0.301	0.306
922300	500	0.244	0.34	889	0.27	0.283
1004400	500	0.28	0.334	777	0.265	0.274
1082500	503	0.221	0.312	783	0.278	0.304
1167700	500	0.248	0.314	861	0.257	0.311
1248300	500	0.252	0.27	769	0.267	0.243
1329000	500	0.25	0.308	798	0.277	0.303
1418300	500	0.266	0.308	835	0.281	0.277
1501600	501	0.206	0.325	781	0.243	0.328
1588300	500	0.276	0.268	826	0.278	0.245
1675200	500	0.216	0.312	778	0.256	0.293
1761300	501	0.226	0.339	795	0.249	0.314
1844300	500	0.216	0.296	798	0.227	0.293
1928500	500	0.192	0.306	782	0.234	0.284
