# Supplementary material for: ChIP on SNP-chip for genome-wide analysis of human histone H4 hyperacetylation
Source: BMC Genomics. 2007 Sep 14;8:322. doi: 10.1186/1471-2164-8-322 (PMC2194786; doi:10.1186/1471-2164-8-322)
Supplement: Additional file 2 — Supplementary Table S2. Genomic correlation gene expression. For all pairs of Affymetrix DNA microarray HGU133A/B probesets detecting transcripts with transcription starts at a given distance range, the table shows the number of pairs according to their detection status. Columns are: distance, upper limit of distance range (in bp); total_blast, number of pairs at the distance range for the myoblast sample; aa_blast, fraction of pairs where both probesets did not detect gene expression in the myoblast sample; pp_blast, fraction of pairs where both probesets detected gene expression in the myoblast sample; total_tube, number of pairs at the distance range for the myotube sample; aa_tube, fraction of pairs where both probesets did not detect gene expression in the myotube sample; pp_tube, fraction of pairs where both probes detected gene expression in the myotube sample. This data was used for the graphs in Figure 2B. [file 1471-2164-8-322-S2.pdf]

Supplementary Table S2

distance	total_blast	aa_blast	pp_blast	total_tube	aa_tube	pp_tube
200	7093	0.443	0.244	7079	0.437	0.266
23100	32110	0.516	0.096	32398	0.517	0.102
60800	41049	0.495	0.1	41190	0.496	0.101
103200	42523	0.495	0.095	42197	0.501	0.093
145200	39490	0.501	0.09	39399	0.51	0.089
192100	42038	0.501	0.091	42119	0.508	0.088
241200	42736	0.493	0.093	42709	0.496	0.092
291400	42496	0.505	0.089	42552	0.509	0.086
335600	36645	0.497	0.09	36847	0.503	0.088
387000	41670	0.499	0.09	41867	0.503	0.089
440000	41922	0.506	0.087	41933	0.508	0.086
488400	38213	0.509	0.085	38141	0.515	0.084
537200	37055	0.51	0.086	37092	0.512	0.084
592700	43077	0.501	0.088	42882	0.505	0.088
645800	40117	0.508	0.084	40157	0.511	0.084
699500	39791	0.509	0.085	39855	0.512	0.085
750400	37649	0.503	0.084	37569	0.508	0.082
800800	37492	0.504	0.088	37375	0.505	0.088
853200	38306	0.502	0.087	38683	0.506	0.086
903200	36648	0.501	0.088	36658	0.501	0.087
955000	36888	0.502	0.088	36864	0.505	0.085
