# Supplementary material for: ChIP on SNP-chip for genome-wide analysis of human histone H4 hyperacetylation
Source: BMC Genomics. 2007 Sep 14;8:322. doi: 10.1186/1471-2164-8-322 (PMC2194786; doi:10.1186/1471-2164-8-322)
Supplement: Additional file 3 — Supplementary Table S3. Association between histone H4 hyperacetylation and gene expression in human myoblasts and myotubes. For all pairs of SNP-array probes and gene transcription starts at a given distance range, the table shows the number of pairs of SNP-array probes and DNA microarray probesets according to their detection status. Columns are: distance, upper limit of distance range (in bp); pT_blast, number of pairs where the SNP-array probe detected histone H4 hyperacetylation and the DNA microarray probeset detected gene expression in the myoblast sample; pS_blast, number of pairs where the SNP-array probe detected histone H4 hyperacetylation and the DNA microarray probeset did not detect gene expression in the myoblast sample; aT_blast, number of pairs where the SNP-array probe did not detect histone H4 hyperacetylation and the DNA microarray probeset detected gene expression in the myoblast sample; aS_blast, number of pairs where the SNP-array probe did not detect histone H4 hyperacetylation and the DNA microarray probeset did not detect gene expression in the myoblast sample; pT_tube, number of pairs where the SNP-array probe detected histone H4 hyperacetylation and the DNA microarray probeset detected gene expression in the myotube sample; pS_tube, number of pairs where the SNP-array probe detected histone H4 hyperacetylation and the DNA microarray probeset did not detect gene expression in the myotube sample; aT_tube, number of pairs where the SNP-array probe did not detect histone H4 hyperacetylation and the DNA microarray probeset detected gene expression in the myotube sample; aS_tube, number of pairs where the SNP-array probe did not detect histone H4 hyperacetylation and the DNA microarray probeset did not detect gene expression in the myotube sample. This data was used for the graphs in Figure 3. [file 1471-2164-8-322-S3.pdf]

Supplementary Table S3

distance	pT_blast	pS_blast	aT_blast	aS_blast	pT_tube	pS_tube	aT_tube	aS_tube
1000000	394	918	293	638	483	1078	411	868
950000	407	881	292	668	460	1043	349	867
900000	377	948	248	577	433	1111	336	778
850000	449	872	234	632	517	1045	317	897
800000	379	964	213	608	424	1147	313	869
750000	435	966	216	609	485	1134	287	836
700000	400	977	249	683	511	1134	326	873
650000	454	880	223	622	524	1097	332	777
600000	381	922	205	595	449	1083	312	811
550000	408	896	214	593	463	1120	273	769
500000	441	901	248	582	472	1098	299	758
450000	472	846	176	623	557	1053	258	794
400000	386	874	186	515	470	1050	263	785
350000	420	871	197	530	497	1079	287	740
300000	396	903	192	554	504	1054	284	768
250000	403	872	219	533	504	1077	230	703
200000	450	891	166	566	547	1082	189	733
150000	385	829	167	525	468	1017	189	684
100000	387	939	115	501	492	1111	139	669
50000	386	930	113	420	478	1146	109	548
-50000	425	973	110	442	547	1172	93	546
-100000	371	926	138	527	500	1180	156	647
-150000	396	917	149	475	510	1115	197	624
-200000	447	965	194	523	546	1203	208	642
-250000	403	893	181	493	522	1038	213	711
-300000	372	970	186	578	501	1168	228	725
-350000	410	885	214	523	508	1143	256	699
-400000	363	1011	223	576	444	1143	274	800
-450000	378	1005	212	551	509	1199	241	787
-500000	424	977	283	508	505	1147	345	739
-550000	391	900	253	577	446	1093	343	781
-600000	372	923	241	544	459	1051	314	700
-650000	430	921	224	587	498	1139	314	772
-700000	438	898	229	598	479	1120	328	764
-750000	402	1007	241	664	444	1137	335	837
-800000	375	957	246	607	458	1189	354	823
-850000	420	951	246	679	476	1076	322	938
-900000	384	818	230	644	449	1025	315	839
-950000	396	922	273	638	418	1075	348	851
-1000000	453	946	284	664	518	1125	374	844
